# Evaluating an Outpatient Rapid Preoperative Optimization Protocol for Poorly Controlled Graves’ Disease: A Retrospective Cohort Study

**DOI:** 10.7759/cureus.118832

**Published:** 2026-10-30

**Authors:** Hao Wei Tan, Mohammad Zaid Seegoolam, Mohummad Shaan Goonoo, Sabapathy P Balasubramanian, Muhammad Fahad Arshad

**Affiliations:** 1 Diabetes and Endocrinology, Sheffield Teaching Hospitals NHS Foundation Trust, Sheffield, GBR; 2 Endocrinology and Diabetes, Northern General Hospital, Sheffield, GBR; 3 Diabetes and Endocrinology, Northern General Hospital, Sheffield, GBR

**Keywords:** cholestyramine, graves’ disease, hyperthyroidism, lugol’s iodine, outpatient care, preoperative optimization, thyroidectomy, thyroid storm

## Abstract

Background: Thyroidectomy is indicated in relapsed or poorly controlled Graves’ disease, thyroid eye disease, pregnancy, suspected malignancy, and large goiter. Guidelines recommend rendering patients biochemically euthyroid and adequately beta-blocked before surgery to reduce the risk of perioperative thyroid storm, but thionamide therapy commonly requires four to eight weeks to achieve this. Rapid multimodal preparation, used where antithyroid drugs fail or are not tolerated, and surgery cannot wait, has conventionally required a 10- to 14-day admission, with no standardized outpatient alternative described. The Sheffield Perioperative Rapid Optimization in Graves’ Disease (SPROG) protocol was developed locally to achieve rapid biochemical control and was adapted for entirely outpatient delivery during the COVID-19 pandemic in response to hospital bed pressures. This study evaluated its safety and effectiveness as an outpatient regimen.

Materials and methods: A single-center retrospective observational study was conducted at a tertiary endocrine surgery unit. Patients with poorly controlled Graves’ disease who underwent thyroidectomy after outpatient optimization between December 2020 and December 2025 were included. Eligibility required free thyroxine above 30 pmol/L or free triiodothyronine above 10 pmol/L at baseline. The protocol escalates therapy stepwise over approximately 14 days, beginning with propranolol and Lugol’s iodine and adding cholestyramine, propranolol titration, and dexamethasone as required, with thyroid function measured on alternate weekdays. The primary efficacy outcome was achievement of either predefined targets, free thyroxine below 30 pmol/L or free triiodothyronine below 10 pmol/L, at the final preoperative assessment; the primary safety outcome was perioperative thyroid storm.

Results: Twenty-three patients were included, 20 (87%) female, with a median age of 41 years (interquartile range 31-47 years). All underwent total thyroidectomy, and none required preoperative admission. Both targets were achieved at the final preoperative assessment in 23 of 23 patients (100%). Median free thyroxine decreased from 78.6 pmol/L at baseline to 24.1 pmol/L (interquartile range 18.25-35.05 pmol/L), and median free triiodothyronine from 38.5 pmol/L to 6.5 pmol/L (interquartile range 5.32-7.58 pmol/L). No perioperative thyroid storm occurred (0 of 23; 95% confidence interval 0 to 14.8%). Off-protocol rescue therapy was required in one patient (4.3%). Hypocalcemia occurred in nine patients (39.1%), was transient in seven (30.4%), and persisted beyond six months in two (8.7%). Voice change occurred in two patients (8.7%) and was long-term in one (4.3%). There were no cases of bleeding, infection, reoperation, readmission, or death. Median postoperative stay was one day. Incidental papillary microcarcinoma was found in five patients (21.7%).

Conclusions: Structured rapid preoperative optimization was delivered entirely on an outpatient basis, with predefined biochemical targets achieved in most patients and no perioperative thyroid storm observed. The retrospective, uncontrolled design and small cohort mean this study cannot establish the safety of the approach with confidence or demonstrate equivalence to inpatient optimization. Prospective comparative evaluation is required before wider adoption.

## Introduction

Graves’ disease is an autoimmune thyroid disorder characterized by the production of thyroid-stimulating hormone receptor antibodies, which bind to and activate the thyrotropin receptor, resulting in unregulated synthesis and secretion of thyroxine and triiodothyronine [1]. This leads to a hypermetabolic clinical state marked by weight loss, palpitations, heat intolerance, and tremor. The disease is the most common cause of hyperthyroidism in iodine-replete areas, with a reported incidence of around 25 per 100,000 adults per year in the United Kingdom; females are affected more commonly than males [2].

Antithyroid drugs are the initial standard treatment, usually given for between six and 18 months using a block-and-replace or titration approach, supported by beta-blockers to control adrenergic symptoms [3]. Adjunctive treatments, such as short-term iodine solutions or glucocorticoids, may be used in situations requiring rapid biochemical control, including preoperative preparation or in patients with severe thyrotoxicosis [3].

Definitive treatment with surgery or radioiodine ablation may be required for relapsed or poorly controlled disease. In patients with relapsed Graves’ disease, practice in the United Kingdom with regard to the adoption of radioiodine ablation, surgery, or a second course of antithyroid drugs is variable [4]. In our center, both surgery and radioiodine are commonly used and are similarly effective [2]. While patient satisfaction rates after both treatments are high, surgery has been associated with better satisfaction rates than radioiodine ablation [5].

Thyroidectomy is also preferred in patients with moderate or severe thyroid eye disease, in pregnancy, in those with coexisting primary hyperparathyroidism, where there is radiological suspicion of malignant thyroid nodules, or in the presence of a large goiter [6].

The primary complications of thyroid surgery are laryngeal nerve injury and hypoparathyroidism, whereas uncommon complications include postoperative bleeding and infection [2]. In preparation for thyroid surgery, the American Thyroid Association guidelines recommend rendering the patient euthyroid and effectively beta-blocked in order to decrease the risk of intraoperative thyroid crisis [7,8]. Thyroid storm is a rare but severe complication of thyrotoxicosis with a high mortality rate [9]. The risk of perioperative thyroid storm is reduced by adequate preoperative optimization with appropriate medical treatment, and various strategies have been proposed to achieve this [9-15]. In patients with uncontrolled disease and those who need urgent surgery, rapid preoperative preparation can be achieved using a combination of drugs including thionamides, cholestyramine, beta-blockers, glucocorticoids, and oral iodine in the form of Lugol’s solution [8,16].

The Sheffield Perioperative Rapid Optimization in Graves’ Disease (SPROG) protocol is a locally developed strategy designed to facilitate the rapid optimization of patients with poorly controlled thyroid disease prior to surgical intervention [16]. While traditionally employed as an inpatient regimen, constraints on hospital capacity and increased bed pressure during the COVID-19 pandemic necessitated adaptation of this protocol to an outpatient model [17,18].

This study, therefore, aimed to assess the safety and efficacy of the protocol when implemented in an outpatient setting for patients with Graves’ disease undergoing rapid optimization prior to surgery. We specifically evaluated the proportion of patients achieving the predefined biochemical targets of free thyroxine below 30 pmol/L or free triiodothyronine below 10 pmol/L at the final preoperative assessment, which constituted the primary efficacy outcome, and the incidence of perioperative thyroid storm, which constituted the primary safety outcome.

## Materials and methods

Study design and setting

A retrospective observational study was conducted at Sheffield Teaching Hospitals NHS Foundation Trust, Sheffield, England, to evaluate the safety and efficacy of the SPROG protocol. Patients who underwent optimization using the protocol between December 2020 and December 2025 were included.

Participants

The study cohort included all patients with Graves’ disease who required thyroidectomy and underwent preoperative optimization using the protocol. Patients were selected for the protocol by the clinical team on the basis of poorly controlled Graves’ disease where they had been intolerant of or resistant to standard antithyroid medication and radioiodine ablation was considered neither feasible nor appropriate, or where surgical intervention was indicated but standard outpatient optimization was insufficient or unfeasible.

For the purposes of this study, poorly controlled disease was defined as free thyroxine above 30 pmol/L or free triiodothyronine above 10 pmol/L, and antithyroid drug intolerance was defined as documented neutropenia, deranged liver function, or rash prompting discontinuation. To be eligible for inclusion, patients were required to have biochemically uncontrolled hyperthyroidism at baseline, defined as free thyroxine greater than 30 pmol/L or free triiodothyronine greater than 10 pmol/L. The same threshold governed escalation of therapy at each subsequent review, as shown in Figure 1.

**Figure 1:**
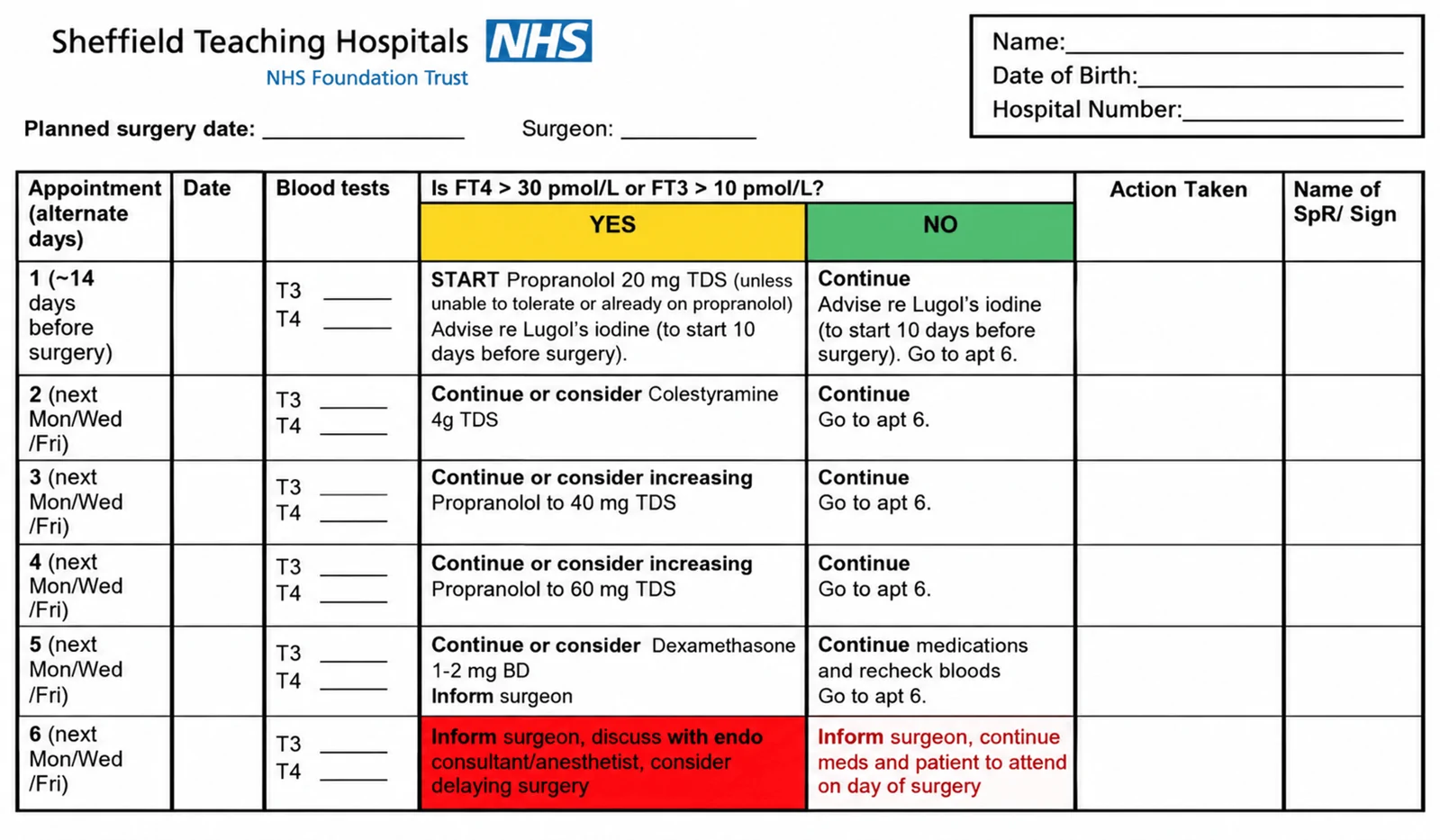
The Sheffield Perioperative Rapid Optimization in Graves’ Disease Protocol TDS: three times daily; BD: twice daily; fT3: free triiodothyronine; fT4: free thyroxine; SpR: specialist registrar. Lithium is not part of the protocol shown and was used separately as background or rescue therapy.

The SPROG protocol

The protocol is an outpatient-based rapid optimization regimen designed to prepare patients for thyroidectomy within approximately two weeks, thereby minimizing the need for inpatient admission. The primary biochemical objectives are to ensure declining free thyroid hormone levels and to bring free thyroxine below 30 pmol/L and free triiodothyronine below 10 pmol/L by the time of surgery. The protocol involves sequential escalation of medical therapy combined with frequent biochemical monitoring, usually performed on alternate weekdays (Monday, Wednesday, and Friday), starting approximately 14 days prior to the planned surgery date.

The therapeutic algorithm is illustrated in Figure 1. At baseline, 15 days before surgery, propranolol 20 mg three times daily was started if not already prescribed or contraindicated, and patients were counseled regarding Lugol’s iodine. Agents were added in the following order: Lugol’s iodine 0.3 mL three times daily, started at least 10 days prior to surgery; cholestyramine 4 g three times daily; titration of propranolol to 40 mg three times daily and then to 60 mg three times daily; and dexamethasone 1-2 mg twice daily.

Escalation to the next agent in this sequence occurred at each alternate-weekday review at which the escalation criterion, free thyroxine above 30 pmol/L or free triiodothyronine above 10 pmol/L, remained met. Where the criterion was not met, existing therapy was continued unchanged, and thyroid function was rechecked at the next scheduled review.

A safety alarm criterion was built into the protocol to inform the operating surgeon if biochemical targets were not met by the final preoperative assessment, between two days before surgery and the day of surgery, allowing a documented discussion regarding the safety of proceeding with anesthesia. Vitamin D levels were checked in all patients, and replacement therapy was initiated before surgery in those identified as deficient.

Background and rescue therapy

Lithium is not part of the protocol. Four patients (17.4%) were already established on lithium carbonate before protocol entry. One patient (4.3%) received lithium carbonate as rescue therapy, commenced during the optimization period outside the defined escalation sequence. This patient had recurrent Graves’ disease that had not responded to antithyroid drug therapy and had markedly deranged thyroid function at protocol entry. Lithium carbonate 300 mg four times daily was commenced on the fifth day of the protocol and continued for 11 days, to the day of surgery. The serum lithium concentration measured two days before surgery was 0.4 mmol/L. The decision to initiate rescue therapy was made by the supervising endocrinology consultant. Background and rescue lithium are reported separately from protocol-defined agents throughout. The patient who received rescue therapy achieved both predefined biochemical targets; the one patient who did not achieve the primary efficacy outcome received no rescue therapy.

Patient management and monitoring

All patients were advised to attend the Endocrine Investigation Unit on alternate days for two weeks prior to thyroidectomy for thyroid function tests and were advised on changes to antithyroid medication. At each visit, patients were reviewed by an endocrinology specialist registrar or a specialist endocrine nurse for repeat thyroid function tests, and for assessment of medication compliance, dose adjustment, and side effects. Surgery was performed approximately 14 days after starting treatment, and treatment was continued until the day of surgery in all patients.

Biochemical assays

Thyroid-stimulating hormone, free thyroxine, and free triiodothyronine were quantified. The reference range for free thyroxine was 11.9-21.6 pmol/L and for free triiodothyronine was 3.1-6.8 pmol/L. Both were measured using competitive electrochemiluminescence immunoassays on cobas e platforms (Roche Diagnostics, Basel, Switzerland), with traceability to equilibrium dialysis-standardized reference methods. Concentrations exceeding the upper reporting limit of the assay were returned by the laboratory as greater than that limit rather than as a numerical value, and were carried through the analysis in that form without imputation or dilution-based extrapolation; consequently, some quartile boundaries are presented with a greater-than notation.

Outcome definitions

The primary efficacy outcome was achievement of either predefined biochemical targets, free thyroxine below 30 pmol/L or free triiodothyronine below 10 pmol/L, at the final preoperative assessment, defined as the last thyroid function measurement obtained before surgery. Achievement of each target individually was reported as a secondary outcome, as was the daily trend in median hormone concentrations. The primary safety outcome was perioperative thyroid storm, ascertained by documented clinical diagnosis. Hypocalcemia was defined as adjusted serum calcium less than 2.10 mmol/L, and was classified as permanent where it persisted beyond six months. Voice change was defined as patient-reported alteration in voice quality, assessed by clinical enquiry and laryngoscopy, and classified as long-term where it persisted at six months.

Surgical and postoperative management

The surgical procedures were performed or supervised by one of two experienced endocrine surgeons. Patients were admitted for an overnight stay after the procedure and had serum calcium and parathyroid hormone levels measured the day after surgery. In addition, all patients underwent voice, wound, and swallowing assessment for postoperative complications. All antithyroid medications were stopped

immediately after surgery, except for propranolol, which was weaned off over two to 14 days in some patients. Oral thyroxine was started at a dose of 1.6 mcg/kg body weight from the morning after surgery.

Data collection, visualization, and analysis

Demographic and clinical data were collected retrospectively. Categorical data are reported as frequencies and continuous data as medians with interquartile ranges. All patient details were accessed only by members of the clinical team, and identifiable details were anonymized before analysis. Data visualization was performed using Python (Python Software Foundation, Wilmington, DE, US) with the pandas and Matplotlib libraries.

The confidence interval for the zero-event safety outcome was calculated using the exact (Clopper-Pearson) binomial method. Biochemical sampling occurred on alternate weekdays, and patients entered the protocol at slightly differing intervals before surgery, so the number of patients contributing a measurement varies by day. Day-level medians are therefore calculated on the patients sampled on that day rather than on the full cohort, and no imputation was performed for days on which a patient was not sampled.

Ethical considerations

This study was a retrospective evaluation of routine clinical care using anonymized data collected as part of standard practice. In accordance with UK Health Research Authority criteria, research ethics committee review was not required for work of this nature, and the requirement for individual informed consent was waived.

## Results

Patient demographics and baseline characteristics

Twenty-three patients were included in the final analysis. The cohort was predominantly female (87%, n=20), with a median age of 41 years (interquartile range 31-47 years). All patients underwent total thyroidectomy, and no patient required inpatient admission before surgery.

Baseline clinical characteristics are presented in Table 1. The median duration of Graves' disease at referral was 27 months (interquartile range 85-43.0 months, range 1-152 months). Twenty-one patients (91.3%) had received antithyroid drug therapy before protocol entry; of these, 15 (65.2%) had failed to achieve control despite treatment, five (21.7%) had discontinued because of intolerance, and one (4.3%) had relapsed six years after an initially successful course. An adverse effect attributed to antithyroid drug therapy was documented in 12 patients (56.5%), of which neutropenia was the most common, occurring in eight (34.8%). Radioiodine had been offered or considered in 14 patients (60.9%) and was precluded by the presence of young children at home in six (26.1%). Thyroid eye disease was documented in eight patients (34.8%), mild in six and moderate in one. A goiter was documented in 13 patients (56.5%) and a thyroid nodule in one (4.3%). Cardiovascular comorbidity was uncommon, comprising atrial fibrillation in one patient (4.3%) and left ventricular hypertrophy in one (4.3%)

**Table 1:** Baseline characteristics of the cohort Percentages are of the full cohort of 23 patients. Indication categories and adverse effect categories are not mutually exclusive, since several patients had more than one. Duration of Graves’ disease is from diagnosis to referral for protocol-based optimization.

Characteristics	n	%
Duration of Graves’ disease at referral		
Median months (interquartile range 8.5-43.0 months; range 1-152 months)	27	-
Indication for thyroidectomy (not mutually exclusive)		
Poorly controlled or refractory disease	23	100.0
Relapsed or recurrent disease	5	21.7
Antithyroid drug intolerance	13	56.5
Thyroid eye disease	8	34.8
Preceding thyroid storm	2	8.7
Previous antithyroid drug therapy		
Any antithyroid drug exposure before protocol entry	21	91.3
Failed to achieve control despite treatment	15	65.2
Discontinued because of intolerance	5	21.7
Initial control achieved, later relapsed	1	4.3
No recorded antithyroid drug exposure	2	8.7
Adverse effects attributed to antithyroid drugs (not mutually exclusive)		
Any adverse effect	13	56.5
Neutropenia	8	34.8
Rash	2	8.7
Gastrointestinal intolerance	1	4.3
Lymphadenopathy	1	4.3
Mouth ulceration	1	4.3
Transaminitis	1	4.3
Unspecified intolerance	1	4.3
Radioiodine		
Offered or considered	14	60.9
Precluded by young children at home	6	26.1
Not offered	2	8.7
Not recorded	1	4.3
Thyroid eye disease		
Absent	14	60.9
Mild	6	26.1
Moderate	1	4.3
Present, severity not recorded	1	4.3
Not recorded	1	4.3
Goiter and nodular status		
Goiter documented	13	56.5
No goiter documented	7	30.4
Goiter status not recorded	3	13.0
Thyroid nodule documented	1	4.3
Cardiovascular comorbidity		
Atrial fibrillation	1	4.3
Left ventricular hypertrophy	1	4.3
None recorded	21	91.3

Therapeutic regimen utilization

Most patients required Lugol’s iodine, cholestyramine, and propranolol. The utilization of specific therapeutic agents is presented in Table 2, grouped by protocol-defined agents, pre-existing background antithyroid therapy, and off-protocol rescue therapy. All patients receiving antithyroid drugs were already established on these medications, with the exception of two patients; in one case, carbimazole was switched to propylthiouracil prior to initiation of the protocol. Rescue therapy outside the protocol-defined escalation sequence was required in one patient (4.3%). Twenty-two of 23 patients (95.7%) were, therefore, managed to the final preoperative assessment using the protocol sequence together with their established background therapy alone.

**Table 2:** Therapeutic regimen utilization Grouped by protocol-defined agents, pre-existing background antithyroid therapy, and off-protocol rescue therapy. Where propranolol was not tolerated, bisoprolol was given.

Group	Therapeutic Agent	Number of Patients (n)	Percentage (%)
Protocol-defined agents	Aqueous iodine solution	23	100.0
	Cholestyramine	20	87.0
	Propranolol	18	78.3
	Dexamethasone	10	43.5
	Bisoprolol	2	8.7
Pre-existing background antithyroid therapy	Carbimazole	6	26.1
	Propylthiouracil	5	21.7
	Lithium	4	17.4
Off-protocol rescue therapy	Lithium	1	4.3

Achievement of biochemical targets

At the final preoperative assessment, free thyroxine below 30 pmol/L was achieved in 16 of 23 patients (69.6%), free triiodothyronine below 10 pmol/L in 21 of 23 (91.3%), and both targets simultaneously in 15 of 23 (65.2%). Only one patient did not achieve the primary efficacy outcome, and the biochemical levels of free thyroxine and free triiodothyronine were 11.3 pmol/L and 30.6 pmol/L, respectively. This patient had completed the full escalation sequence, receiving propranolol, Lugol's iodine, cholestyramine, and dexamethasone. In accordance with the protocol's safety alarm criterion, the operating surgeon was informed, and a documented discussion took place, after which the patient was considered appropriate to proceed. Surgery was undertaken as planned, and no perioperative thyroid storm or other major adverse event occurred.

Biochemical trends

Median free thyroxine decreased from 78.6 pmol/L (interquartile range 71.8 to greater than 100.0 pmol/L) 15 days prior to surgery to 24.1 pmol/L (interquartile range 18.25-35.05 pmol/L) on the day before surgery. Over the same period, median free triiodothyronine decreased from 38.50 pmol/L (interquartile range 30.4 to greater than 50 pmol/L) to 6.5 pmol/L (interquartile range 5.32-7.58 pmol/L). The trend is shown in Figure 2, and the values are listed in Table 3.

**Figure 2:**
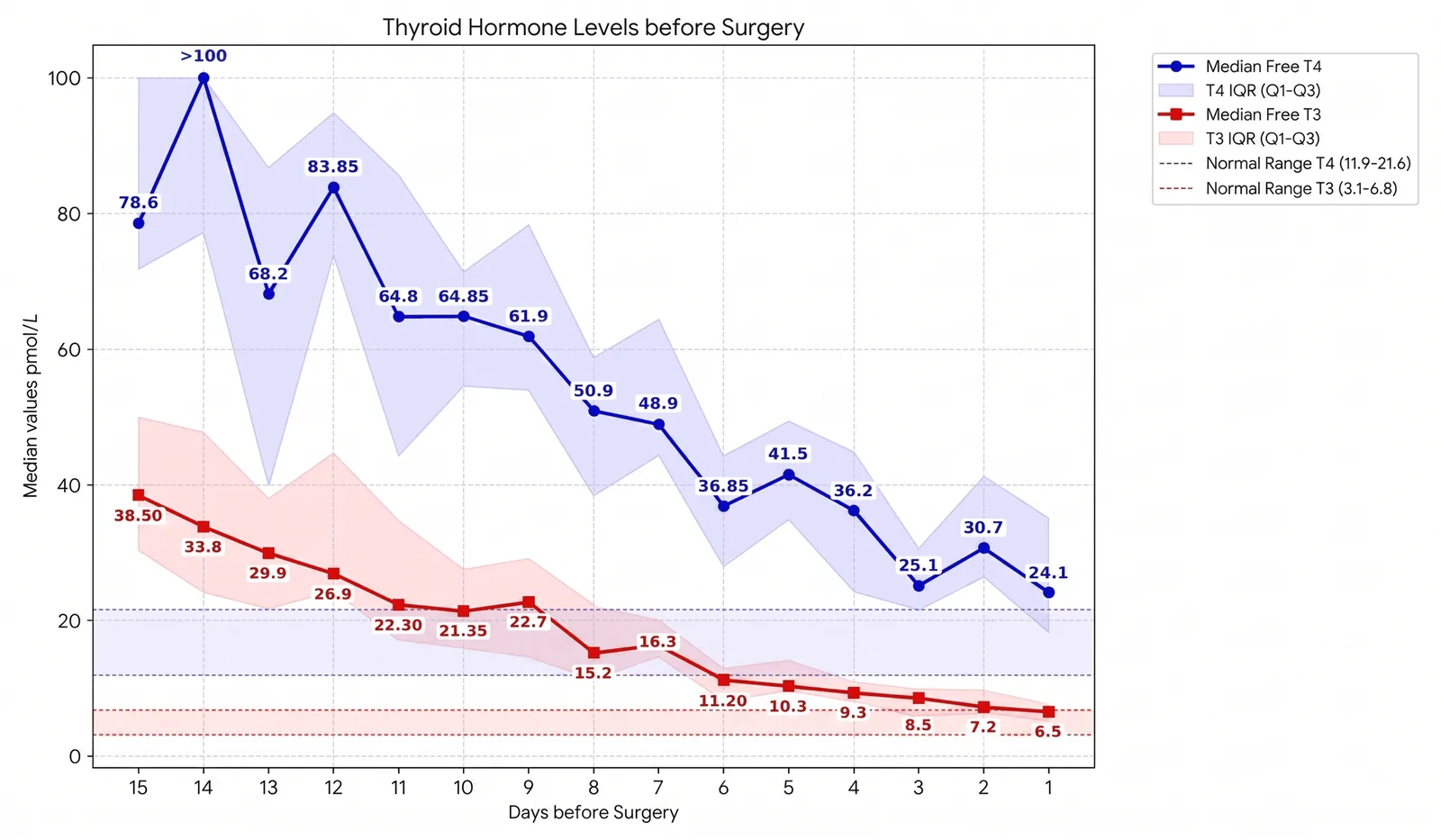
Thyroid hormone levels before surgery T4: thyroxine; T3: triiodothyronine; IQR: interquartile range; Q1: first quartile; Q3: third quartile. The figure shows cohort-level medians computed from the patients sampled on each day, not a within-patient trajectory, and that the number of contributing patients varies by day.

**Table 3:** Summary of thyroid hormone levels before surgery fT4: free thyroxine; fT3: free triiodothyronine; Q1: first quartile; Q3: third quartile. Values above the assay reporting limit are shown with a greater-than notation. The number of patients contributing a measurement varies by day because sampling occurred on alternate weekdays

Day	Median Free T4 /pmol/L	Q1	Q3	Number of Patients	Median Free T3 /pmol/L	Q1	Q3	Number of Patients
15	78.6	71.8	>100.0	5	38.50	30.4	>50	5
14	>100	77.25	>100.0	8	33.8	24.15	47.7	7
13	68.2	39.85	86.8	11	29.9	21.7	38.0	11
12	83.85	73.78	94.9	8	26.9	24.35	44.68	6
11	64.8	44.3	85.7	9	22.30	17.1	34.70	7
10	64.85	54.55	71.47	8	21.35	15.95	27.5	8
9	61.9	53.98	78.35	8	22.7	14.6	29.1	8
8	50.9	38.4	58.8	13	15.2	11.32	22.28	12
7	48.9	44.35	64.45	11	16.3	14.65	20.02	10
6	36.85	27.98	44.28	14	11.20	8.12	12.88	14
5	41.5	34.9	49.4	11	10.3	9.65	14.12	10
4	36.2	24.25	44.85	7	9.3	8.0	10.9	7
3	25.1	21.55	30.62	12	8.5	5.75	9.9	11
2	30.7	26.42	41.3	10	7.2	6.4	9.7	9
1	24.1	18.25	35.05	16	6.5	5.32	7.58	16

Free thyroxine and free triiodothyronine levels showed a consistent downward trend as patients approached the day of surgery. Free triiodothyronine levels normalized to within the reference range by the day before surgery, whereas free thyroxine levels achieved the protocol-defined target of less than 30 pmol/L. Variation in the data narrowed over time, indicating more stable and more consistent hormone levels across the patient group as surgery approached.

Safety outcome

No patient developed perioperative thyroid storm (0 of 23; 0%, 95% confidence interval 0 to 14.8%).

Histological outcomes

Postoperative histological analysis confirmed Graves’ disease alone in 69.6% (n=16) of patients, with incidental papillary microcarcinoma identified in 21.7% (n=5). Diffuse thyroid hyperplasia and follicular nodular disease were identified in one patient each.

Complications

Hypocalcemia occurred in nine patients (39.1%), of which the majority were transient (n=7, 30.4%). Long-term hypocalcemia, persisting beyond six months and requiring long-term vitamin D and calcium replacement, occurred in two patients (8.7%). Voice changes were observed in two patients (8.7%). Of these, one patient (4.3%) experienced long-term changes requiring ongoing voice rehabilitation, while the other experienced only transient changes. There were no cases of postoperative bleeding, infection, reoperation for hematoma or hemorrhage, or readmission.

Postoperative complications are presented in Table 4. There were no deaths during the follow-up period. The median length of stay after surgery was one day (interquartile range 1-1 day).

**Table 4:** Postoperative complications

Complication	Status	Number of Patients (n)	Percentage
Hypocalcemia	Total incidence	9	39.1
	Transient	7	30.4
	Long-term replacement	2	8.7
Voice changes	Total incidence	2	8.7
	Transient/Recovered	1	4.3
	Long-term (requiring rehabilitation)	1	4.3

## Discussion

The SPROG protocol was developed as a pragmatic, outpatient-based approach to preoperative optimization, particularly in the context of increasing hospital bed pressures during the COVID-19 pandemic and the need for timely surgery. In this cohort, outpatient delivery achieved the predefined biochemical targets in most patients within approximately 14 days, and no perioperative thyroid storm was observed.

Current literature on preoperative optimization

Preoperative optimization in Graves’ disease is essential to achieve a biochemically euthyroid state and to prevent the potentially fatal complication of thyroid storm during or after thyroidectomy [6]. Despite this, there is no universally standardized protocol for the rapid optimization of patients with poorly controlled disease or those requiring urgent surgery. Current international guidelines, such as those from the American Thyroid Association, primarily recommend an approach using thionamides such as carbimazole or propylthiouracil, which often require four to eight weeks to achieve a euthyroid state [7]. Adjunctive measures are standardized, and beta-adrenergic blockade, potassium iodide, glucocorticoids, and potentially cholestyramine are recommended in the immediate preoperative period. However, the literature lacks a clear, structured protocol for rapid optimization. The accepted approach for refractory hyperthyroidism typically involves immediate hospital admission for intensive monitoring and multimodal drug escalation, with no emphasis on the possibility of an outpatient approach.

Biochemical response

The cohort was characterized by prolonged and refractory disease: the median duration of Graves’ disease at referral was more than two years, an adverse effect attributable to antithyroid drugs was documented in over half the cohort, and radioiodine was precluded by childcare commitments in approximately a quarter. These features support the description of this population as high risk and should be borne in mind when comparing the complication rates reported here with those from unselected surgical series.

The predefined targets were achieved in most patients within approximately 14 days, offering a potential operational advantage over conventional optimization, which typically requires inpatient admission or prolonged outpatient management. Although the final median free thyroxine remained slightly above the upper reference limit, it lay within the protocol’s target of less than 30 pmol/L, and the median free triiodothyronine normalized within the laboratory reference range. These findings should be interpreted as the response of a cohort receiving a multimodal strategy, comprising the protocol sequence together with established background antithyroid therapy and, in one patient, rescue therapy, rather than as evidence of the independent effect of the escalation sequence.

Aqueous iodine solution was used in all 23 patients (100.0%), consistent with its established role in decreasing thyroid vascularity and inhibiting hormone release via the Wolff-Chaikoff effect [7]. Propranolol was used in 78.3% (n=18) of cases, with 8.7% (n=2) receiving bisoprolol. These agents provide essential symptomatic control and inhibition of peripheral conversion [7]. Cholestyramine was used in 87.0% (n=20) of patients, functioning as a bile acid sequestrant that interferes with the enterohepatic circulation of thyroid hormones and is a particularly effective adjunct in severe thyrotoxicosis [7]. This effect has also been shown to be efficacious and well tolerated in a separate study [19]. Furthermore, the use of dexamethasone in 43.5% (n=10) of the cohort facilitated inhibition of peripheral thyroxine to triiodothyronine conversion and suppressed thyroid hormone secretion [7].

Lithium inhibits the release and synthesis of thyroid hormones and is occasionally used alongside radioiodine ablation to treat Graves’ disease [20] and has been shown to be beneficial in achieving a euthyroid state in refractory hyperthyroidism [21]. In this cohort, it was used predominantly as established background therapy in patients unable to tolerate thionamides and as rescue therapy in a single patient. The high rate of adjunctive therapy overall reflects the complex clinical baseline of this cohort, in which standard thionamide therapy was insufficient, poorly tolerated, or contraindicated.

Surgical outcomes

The overall incidence of postoperative hypocalcemia was 39.1% (n=9); however, only 8.7% (n=2) of patients had hypocalcemia persisting beyond six months and requiring long-term calcium and vitamin D supplementation (Table 4). Similarly, the rate of long-term voice change was low (4.3%, n=1). Despite the high-risk patient profile, the observed surgical complication rates were consistent with those reported in the existing literature. A retrospective study examining postoperative complications following thyroidectomy for Graves’ disease reported overall incidences of postoperative hypocalcemia and long-term voice change of 12.4% and 0.9%, respectively, although the definition of hypocalcemia was not standardized [22]. As that study included both emergency and elective thyroidectomy cases, the reported complication rates may be lower than those in cohorts consisting predominantly of higher-risk surgical populations.

Incidental papillary microcarcinoma was identified in 21.7% of this cohort. This is higher than figures reported elsewhere; thyroid cancer has been reported in up to 12.7% of patients with Graves’ disease undergoing surgery without preoperative suspicion of malignancy [23]. This difference may be a chance finding in a small series or may reflect the selected, high-risk nature of a cohort referred for surgery because of refractory or complex disease, and it cannot be generalized to the wider Graves’ population. The observation is nonetheless a reminder that Graves’ disease does not preclude coexisting thyroid malignancy and warrants confirmation in larger unselected series.

Operational impact

Delivering the protocol on an outpatient basis avoided preoperative admission in all 23 patients. Conventional rapid optimization for high-risk thyrotoxicosis is typically undertaken during an inpatient stay of 10 to 14 days, and avoiding such admissions has the potential to release acute capacity at a time of high bed occupancy across the National Health Service [24,25]. We did not undertake a formal cost-effectiveness analysis, and no comparator admission was observed within this cohort, so the financial consequences of this shift cannot be quantified from these data.

Strengths and limitations

This study describes the feasibility and biochemical outcomes of a structured outpatient protocol for rapid preoperative optimization in Graves’ disease, addressing a setting in which inpatient admission has traditionally been required. Its limitations are substantial. The design is retrospective, observational, and single-center, and the sample is small, which limits generalizability and statistical power. The absence of a comparator group means that no comparison with inpatient optimization is possible, and non-inferiority to inpatient care is an assumption rather than a finding. The small sample precludes meaningful conclusions about rare safety outcomes: no perioperative thyroid storm was observed, but the upper bound of the 95% confidence interval around this zero-event finding does not exclude an event rate of approximately one in seven. Patients received heterogeneous background antithyroid therapy and one received rescue therapy outside the protocol, so the reported outcomes reflect a multimodal strategy rather than the escalation sequence in isolation, and the independent contribution of the protocol cannot be determined. Surgery was performed or supervised by two experienced endocrine surgeons in a high-volume specialist center, and the complication rates may not be reproducible in units with different case volumes. No formal cost-effectiveness analysis was undertaken.

## Conclusions

Structured rapid preoperative optimization was delivered entirely on an outpatient basis in a cohort with poorly controlled Graves’ disease, with no perioperative thyroid storm or major adverse events observed and complication rates consistent with published surgical series. These findings support the feasibility of outpatient delivery and suggest that routine preoperative admission may not be necessary for all such patients. However, the retrospective, uncontrolled design and the small cohort mean that this study cannot establish the safety of the approach with confidence, nor demonstrate equivalence to inpatient optimization, nor isolate the contribution of the protocol from that of background and rescue therapy. Prospective comparative evaluation with predefined outcomes is required before wider adoption can be recommended.
